# How to decrease bronchopulmonary dysplasia in your neonatal intensive care unit today and “tomorrow”

**DOI:** 10.12688/f1000research.10832.1

**Published:** 2017-04-21

**Authors:** Leif D. Nelin, Vineet Bhandari

**Affiliations:** 1 Section of Neonatology, Department of Pediatrics, Nationwide Children’s Hospital, The Ohio State University College of Medicine, Columbus, OH, USA; 2 Section of Neonatology, Department of Pediatrics, St. Christopher’s Hospital for Children, Drexel University College of Medicine, Philadelphia, PA, USA

**Keywords:** bronchopulmonary dysplasia, BPD, neonatal intensive care

## Abstract

Bronchopulmonary dysplasia, or BPD, is the most common chronic lung disease in infants. Genetic predisposition and developmental vulnerability secondary to antenatal and postnatal infections, compounded with exposure to hyperoxia and invasive mechanical ventilation to an immature lung, result in persistent inflammation, culminating in the characteristic pulmonary phenotype of BPD of impaired alveolarization and dysregulated vascularization. In this article, we highlight specific areas in current management, and speculate on therapeutic strategies that are on the horizon, that we believe will make an impact in decreasing the incidence of BPD in your neonatal intensive care units.

## Introduction

Bronchopulmonary dysplasia, or BPD, is the most common chronic lung disease in infants ^1, 2^. Despite many advances in neonatal-perinatal medicine, such as the administration of prenatal steroids, the introduction of surfactant, and “gentler” ventilation strategies, the incidence of BPD—as it has been historically defined—has not declined ^3^. In the USA, the majority of infants developing “new” BPD have a birth weight of <1,250 g, adding 10–15,000 new cases/year ^4^. The burden of this disease, however, is exaggerated, as survivors continue to have pulmonary and neurodevelopmental sequelae, even as adults ^5^.

In this commentary, we have attempted to highlight specific areas in the current management of premature neonates that we believe will make an impact in decreasing the incidence of BPD in your neonatal intensive care units (NICUs). We also speculate on therapeutic strategies that are on the horizon that would potentially further enable the process of continuing that trend.

## Definition of bronchopulmonary dysplasia

BPD currently is most often defined in babies born before 32 weeks using the NICHD/ORD consensus definition, which defines BPD based on need for supplemental oxygen at 28 days of life, and then grades BPD as mild, moderate, or severe depending on supplemental oxygen and respiratory support needs at 36 weeks’ postmenstrual age (PMA) ^6^. For this definition, the severity grades are defined as follows, based on needs at 36 weeks’ PMA: mild BPD is breathing room air, moderate BPD is the need for <30% supplemental oxygen, and severe BPD is requiring ≥30% supplemental oxygen and/or positive pressure ^6^.

There is growing concern that the consensus definition of BPD may fail to adequately classify infants, which has resulted in various modifications to the definition of BPD ^7^, although all of the modified definitions continue to utilize supplemental oxygen and/or respiratory support needs. Recently, the Prematurity and Respiratory Outcomes Program (PROP) investigators examined various definitions for BPD and found that the incidence of BPD in the PROP cohort varied from 32 to 59% depending entirely on which definition of BPD was used ^7^. Furthermore, in this cohort, 2 to 16% of patients could not be classified depending on which definition of BPD was used ^7^. For additional detail and detailed discussion on the definition and epidemiology of BPD, please see a recent review ^8^.

## Pathogenesis of bronchopulmonary dysplasia

BPD occurs as a result of gene–environment interactions ^2, 9^. Genetic predisposition and developmental vulnerability secondary to antenatal and postnatal infections, compounded with exposure to hyperoxia and invasive mechanical ventilation to an immature lung, result in persistent inflammation (and its consequences, e.g. cell death), culminating in the characteristic pulmonary phenotype of BPD of impaired alveolarization and dysregulated vascularization ^10^. For the persistent inflammation and lung remodeling to occur, it does require a sustained duration of exposure to environmental insults ^10^. While the parameters of the early inflammatory response (e.g. cytokines) may not be detectable after prolonged exposure to the above factors, the downstream signaling inflammatory/immune pathways have initiated and affected permanent structural and functional deficits in the BPD lungs, as well borne out by the same being noted in children and adult survivors of BPD ^10– 13^. There is some clinical evidence that early interruption of the initial inflammatory response could result in the amelioration and potential reversal of these effects ^14^.

## How to decrease bronchopulmonary dysplasia today

### Delivery room strategy

In 2010, while the American Heart Association (AHA), the European Resuscitation Council (ERC), and the International Liaison Committee on Resuscitation (ILCOR) issued recommendations that have clearly stated that room air should be used to initiate resuscitation in term infants ^15^, recommendations for preterm infants are still not definitive. As exposure to hyperoxia is a critical factor in the pathogenesis of BPD ^16^, it is important to try and reduce the exposure to high concentrations of supplemental O _2_ as early as possible given the immature anti-oxidant defenses of the preterm newborn ^17^. Pulse oximetry has been used to assess “normal” oxygen saturation (SpO _2_) values after birth in preterm infants, and the median time to achieve SpO _2_ of >80% and >90% was 7.3 and 8.1 minutes, respectively ^18^. Multiple studies have assessed the use of low and high (including titration) concentrations of supplemental O _2_ in the delivery room (DR) ^19– 21^. Meta-analyses have revealed that mortality and other outcomes are not significantly different in preterm infants when starting with a low (≤0.3) or high (≥0.6) fraction of inspired O _2_ (FiO _2_) ^22, 23^. However, given that the overall estimates of effect have a wide range of confidence intervals, additional data are required to be definitive. Currently, we would recommend initiating resuscitation in the DR with a default setting of FiO _2_ of 0.3–0.4 ^24^ and titrating by 5–10% upwards or downwards. Using a T-piece resuscitator to provide continuous positive airway pressure (CPAP) or non-invasive intermittent positive pressure ventilation (NIPPV) is recommended. The blow-by O _2_ should be set at a FiO _2_ of 1.0. During the first few minutes of life, a SpO _2_ of 70–80% may be acceptable, as long as the heart rate is increasing, the baby is ventilating, and the SpO _2_ is increasing. If the SpO _2_ is <85% at 5 minutes, increase the FiO _2_ concentration by 5–10% via the blender. If the SpO _2_ is >93%, gradually decrease the FiO _2_ concentration by 5–10% to maintain the SpO _2_ in the desired range (see below).

### Oxygen supplementation beyond the delivery room

While there is significant ongoing controversy regarding the precise SpO _2_ target ranges to be employed beyond the DR for preterm infants ^25– 29^, we would recommend the lower alarm limit to be 88% and the higher alarm limit to be 96%. Attempting to target SpO _2_ 88–92% would be appropriate ^17, 30, 31^. For the older (>34 weeks) preterm infants on supplemental O _2_ and/or with retinopathy of prematurity stages 2–3 and/or to prevent/manage pulmonary hypertension, we would recommend a target SpO _2_ of 93–97%, with alarm limits of 92–98%.

### Support with non-invasive ventilation

As discussed above, the pathogenesis of BPD includes exposure to mechanical ventilation, suggesting that by avoiding invasive mechanical ventilation, i.e. mechanical ventilation via an endotracheal tube, the risk for developing BPD may be decreased. The SUPPORT trial ^32^ studied 1,316 infants born at <28 weeks’ gestation randomly assigned to intubation and surfactant or nasal CPAP (nCPAP) in the DR and found that the use of nCPAP was a safe alternative to intubation and surfactant in preterm infants. A recent meta-analysis ^33^ comparing prophylactic nCPAP with invasive mechanical ventilation demonstrated that the use of nCPAP resulted in a modest decrease in the risk of developing BPD (relative risk [RR] 0.89, 95% confidence interval [CI] 0.79–0.99, *p*=0.04). Another meta-analysis ^34^ examined avoidance of endotracheal mechanical ventilation and the development of BPD, in which the authors reported a small but significant benefit of avoiding endotracheal mechanical ventilation (i.e. successfully managing with non-invasive respiratory support) on the development of BPD (odds ratio [OR] 0.83, 95% CI 0.71–0.96, *p*=0.01). Detailed discussion on the various modes of non-invasive respiratory support in the NICU for the prevention of BPD has been recently summarized ^35^.

### Extubate early

While avoiding endotracheal mechanical ventilation is associated with less BPD, often in these patients endotracheal mechanical ventilation is necessary. This raises the question of whether earlier extubation decreases the risk of BPD. Robbins *et al.* ^36^ examined 224 patients born at <27 weeks’ gestation and found that the age at first extubation attempt correlated directly with endotracheal mechanical ventilation days and length of stay despite a median mechanical ventilation days of 32 and 65% of patients needing re-intubation. Furthermore, they reported that the earlier an extubation attempt was made, the lower the rate of BPD ^36^. Berger *et al.* ^14^ reported in a cohort of 262 infants born at ≤28 weeks of gestation that the risk of BPD increased when extubation was delayed past the first week of life. In a large retrospective cohort study of extremely low-birth-weight infants (<1,000 g), Jensen *et al.* ^37^ found that the risk of developing BPD increased with duration of mechanical ventilation, but the risk of developing BPD was not related to the number of ventilation courses. Since the number of extubation attempts did not correlate with BPD risk, it should be inferred that reducing the use of mechanical ventilation can be done safely and should reduce the risk for BPD and, furthermore, the sooner an extubation attempt is made, the lower the risk of BPD.

### Role of nutrition

Nutrition plays an important role in the outcomes of preterm infants, and nutritional deficits are likely involved in the pathogenesis of BPD. This raises the question of whether early nutrition has an impact on the incidence of BPD. A recent report using national data from Sweden ^38^ found that having a birth weight classified as small for gestational age (SGA) was a significant risk factor for developing BPD (adjusted OR 2.73, 95% CI 2.11–3.55, *p*<0.05). Wemhöner *et al.* ^39^ reported that preterm infants who went on to develop BPD had significantly ( *p*<0.01) lower cumulative enteral carbohydrates, protein, and calories in the first 14 days of life than did similar preterm infants who did not go on to develop BPD. Ehrenkranz *et al.* ^40^ found in a cohort of 695 infants that as the rate of weight gain increased, the incidence of BPD significantly decreased. Recently, it was reported that, in a cohort of 1,433 very-low-birth-weight (<1,500 g) infants, exclusive formula feeding increased the risk of BPD as compared to exclusive breastmilk feeding (OR 2.59, 95% CI 1.33–5.04, *p*<0.05) ^41^. These association studies support the concept that early nutrition affects the development of BPD and that the provision of good nutrition using breastmilk early can potentially decrease the risk of developing BPD.

### Prevent and/or treat infections

Infection has been identified as an important antecedent to BPD, likely through causing persistent immune regulation in a susceptible preterm infant with other environmental risk factors for BPD ^10^. Indeed, Swedish national data for infants born at ≤32 weeks’ gestation revealed that having one episode of late infection significantly increased the risk of developing BPD (adjusted OR 1.69, 95% CI 1.30–2.21) and having two or more episodes of late infection further increased the risk of developing BPD (adjusted OR 2.69, 95% CI 1.82–3.98) ^38^. A recent report using data from the California Perinatal Quality Care Collaborative found that nosocomial infections increased the risk of developing BPD (OR 2.74, 95% CI 2.54–2.94) and, furthermore, when quality improvement (QI) initiatives resulted in nosocomial infection rates falling, the rates of BPD fell as well ^42^. Kelly *et al.* ^43^ reported in a propensity-matched retrospective cohort study in very-low-birth-weight infants that postnatal cytomegalovirus infection was associated with an increased risk of developing BPD (RR 1.33, 95% CI 1.19–1.50, *p*<0.001). Nosocomial infections (such as rhinovirus in the NICU population) may increase not only the rate but also the severity of BPD ^44^.

Given the role of infection in the development of BPD, consideration has been given to the use of antibiotics in high-risk infants. One organism that has been implicated in the development of BPD is *Ureaplasma*, which has led to the notion that macrolides, particularly azithromycin, may be effective in preventing BPD. Nair *et al.* ^45^ performed a meta-analysis and found three studies examining prophylactic azithromycin and BPD that demonstrated a modest reduction in BPD (RR 0.83, 95% CI 0.71–0.91, number needed to treat=10). However, the authors concluded that the routine use of azithromycin in this population should wait for further studies, including pharmacokinetics and longer-term safety. This is particularly important given recent data showing that antibiotic use in very-low-birth-weight infants may actually increase the risk of developing BPD. For example, Novitsky *et al.* ^46^ found in very-low-birth-weight infants that receiving >48 hours of antibiotics during the first week of life doubled the risk of developing BPD (adjusted OR 2.2, 95% CI 1.4–3.5) and that each additional day of antibiotics beyond the first week of life increased the risk of developing BPD (adjusted OR 1.2 per antibiotic day, 95% CI 1.1–1.2). Similarly, Cantey *et al.* ^47^ recently reported that each additional day of antibiotics in the first 2 weeks of life in infants born at <29 weeks’ gestation significantly increased the risk of developing severe BPD (OR 1.15, 95% CI 1.08–1.27).

### Conclusions

In conclusion for how to decrease BPD today, consideration must be given to the pathogenesis and modifiable pathogenic factors that have supporting evidence. Obviously, prematurity is a significant risk factor for developing BPD, and avoiding preterm delivery will decrease the incidence of BPD. Non-invasive ventilation should be the initial therapy of choice in preterm infants. If a preterm infant has to be intubated, extubation attempts should be done as soon as possible. Extubation should be attempted when there is sufficient spontaneous respiratory effort, the level of mechanical ventilatory support (particularly the peak inspiratory pressures) has been weaned to reasonable levels, and the patient is a suitable candidate for non-invasive mechanical support as a transitional therapy. Excellent nutrition should be provided as early as is feasible, preferably using breastmilk. Finally, prevention of infection using meticulous infection control measures as well as good antibiotic stewardship should be the standard of care for preterm infants. These measures, which are possible today, will decrease the incidence of BPD.

## How to decrease bronchopulmonary dysplasia “tomorrow”

### Newer surfactants

While surfactant treatment in preterm neonates with respiratory distress syndrome (RDS) has not been shown to decrease BPD *per se* ^48, 49^, some newer “enhanced” surfactants are available and are being tested ^50^. No recent data have been published using a surfactant with recombinant surfactant protein (SP)-C (Venticute®) in preterm neonates ^50^. CHF5633 (Chiesi) is a new synthetic surfactant which contains 0.2% SP-B and 1.5% SP-C analogs, along with phospholipids, and has shown some benefit over Survanta® in animal studies ^51^. A phase I/II human study is ongoing.

### Surfactant plus non-invasive intermittent positive pressure ventilation

The combination of using a less-invasive mode of surfactant delivery with non-invasive ventilation strategies appears to be beneficial in decreasing BPD ^52, 53^, though more data are required.

### Steroids

In a double-blinded, randomized, placebo-controlled trial in infants <28 weeks of gestation, low-dose hydrocortisone (HC; 1 mg/kg/day, divided every 12 hours, for 7 days, followed by 0.5 mg/kg/day for 3 days; n=255 analyzed) versus placebo (n=266 analyzed) significantly increased survival without BPD ( *p*=0.04) ^54^. There was a significantly higher rate of extubated patients from 1 week to postnatal day 10 in the HC group. The study noted that older gestational age (26–27 weeks), female sex, and HC treatment were independent factors for survival without BPD, while patent ductus arteriosus ligation and late-onset sepsis increased the risk for BPD/death using logistic regression analysis ^54^. There was a significantly ( *p*=0.02) higher rate of sepsis in the 24–25 weeks’ gestation HC subgroup. The number of patients who would be needed to treat to gain one BPD-free survival was 12 ^54^.

In a placebo-controlled trial, use of inhaled budesonide (two puffs [200 µg/puff] every 12 hours for 14 days, followed by one puff every 12 hours until infant not on supplemental O _2_ or PPV or reached 32 weeks’ PMA; n=437) in infants <28 weeks’ gestation showed a significantly ( *p*=0.05) improved outcome of death or BPD versus placebo (n=419) ^55^.

The second randomized study used placebo (n=104) or fluticasone (two doses every 24 hours [50 µg/dose]; n=107) for 6 weeks or until extubation ^56^. This study did not find any significant difference in the primary outcome or neurodevelopmental outcomes at 3 years but also did not reach the intended sample size ^56^.

### Surfactant plus steroids

In another randomized study, infants (<1,500 g birth weight) with RDS requiring mechanical ventilation and FiO _2_ of ≥0.5 were randomized to receive either surfactant (Survanta®; n=134) alone or surfactant with 0.25 mg/kg (1 ml/kg) of budesonide (n=131) every 8 hours, to a maximum of six doses ^57^. There was a significantly improved primary outcome of less death or BPD, faster weaning to non-assisted O _2_ therapy, and weaning to room air in the surfactant plus steroid group ^57^. There was no significant difference in neurodevelopmental outcomes between the two groups at a mean age of 30 months ^57^.

### Progenitor cells

The risk for abnormal alveolar and airway development in adult survivors of BPD suggests defective development and repair capacity, possibly due to loss of progenitor cells. Data regarding the pre-clinical studies using stem cells to prevent BPD have been recently reviewed ^58^. Phase I human clinical trials have been conducted, and long-term safety and efficacy data continue to be collected ^58^.

### Altering the airway and/or gut microbiome?

Recently, data have been published that suggest fetal acquisition of an airway microbiome in human preterm infants ^59^. Interestingly, *Lactobacillus* was noted to be decreased at birth in infants with chorioamnionitis and those subsequently developing BPD ^59^. Longitudinal alterations in ventilated preterm infants who eventually developed severe BPD revealed greater bacterial community turnover with age, with decreased acquisition of *Staphylococcus* in the first days after birth but increased presence of *Ureaplasma* ^60^. The gut–lung axis could also impact on the immunology of the lung ^61^. Hence, understanding dysbiosis of the airway and gut could potentially allow targeted probiotic therapy to be developed for the prevention of BPD.

### Conclusions

Regarding newer surfactants, if shown to be superior to currently used surfactants, especially if used in combination with less-invasive modes of surfactant administration and non-invasive ventilation strategies ^52^, it may potentially impact on the incidence of BPD in the future. While the low-dose HC study is promising, the number of infants required to be treated is quite close to that of the vitamin A treatment strategy to prevent BPD. The increased risk of infection, albeit in a subgroup, is also of concern. Regarding the inhaled steroid trials, the duration of use is fairly long, with either modest or no benefit. Long-term developmental data are needed for the low-dose HC and inhaled budesonide studies. The use of surfactant as a vehicle to deliver steroids is exciting and, if confirmed with larger trials and combined with less-invasive modes of administration and non-invasive ventilation, could be the next major advance in preventing BPD “tomorrow”. For the “day after tomorrow”, stem cells (or some part of their secretome) and altering the airway microbiome (intra-tracheal delivery of *Lactobacillus* with surfactant?) may be therapeutic approaches to look forward to in the not-too-distant future.
